# Effect of Quinoa (*Chenopodium quinoa* Willd.) Starch and Seeds on the Physicochemical and Textural and Sensory Properties of Chicken Meatballs during Frozen Storage

**DOI:** 10.3390/foods10071601

**Published:** 2021-07-09

**Authors:** Jin-Hwa Park, Yun-Jin Lee, Jeong-Gyu Lim, Ji-Hye Jeon, Ki-Sun Yoon

**Affiliations:** 1 Department of Food and Nutrition, College of Human Ecology, Kyung Hee University, 26 Kyungheedae-ro, Dongdaemun-gu, Seoul 02447, Korea; parkjinhwa@kfri.re.kr (J.-H.P.); cvwpsy93@gmail.com (Y.-J.L.); ijh9460@naver.com (J.-G.L.); lovin_sz@naver.com (J.-H.J.); 2 Korea Food Research Institute, Wanju 55365, Korea

**Keywords:** quinoa, chicken meatballs, freeze-thaw cycles, antioxidant activity, lipid oxidation

## Abstract

The effects of starch (corn and quinoa) and quinoa seeds on chicken meatballs’ physicochemical, textural, and sensory properties were investigated during frozen storage. The chicken meatballs were prepared with corn starch (CS), quinoa starch (QS), quinoa seeds (Q), and combinations of corn starch and quinoa seeds (CS-Q), and quinoa starch and quinoa seeds (QS-Q), which were subjected to five freeze–thaw (F-T) cycles of temperature fluctuation conditions during frozen storage. Regardless of the type used (CS or QS), adding starch resulted in fewer cooking, drip, and reheating losses in chicken meatballs during frozen storage. The values of the hardness, gumminess, and chewiness of chicken meatballs with CS or QS were half those of chicken meatballs without starch, indicating that the addition of starch inhibited the change in the meatballs’ texture. The total volatile basic nitrogen (TVB-N) and thiobarbituric acid reactive substance (TBARS) values were progressive but did not dynamically increase during five F-T cycles. Chicken meatballs containing CS-Q or QS-Q showed significantly lower TBARS values than those with CS, QS, or Q after five F-T cycles. Adding quinoa seeds significantly increased the antioxidant activity and the chewiness of meatballs (*p* < 0.05) compared with starch only. The addition of the combination of QS-Q to chicken meatballs increased the values of taste, texture, and overall acceptability, indicating that quinoa starch and seeds may be introduced as premium ingredients to frozen meat products.

## 1. Introduction 

Frozen ready-to-cook (RTC) meat products are gaining popularity due to their convenience. RTC products require minimal cooking prior to consumption, and cooking is usually undertaken using a conventional grill or a microwave oven at home [1]. Chicken meat is tender and more digestible than red meat. In addition, it has low fat and cholesterol content, high nutritional value, distinct flavor, and abundant proteins (15–20%), as well as essential amino acids, mineral salts, and vitamins [2]. Chicken meatballs are popular frozen RTC products and comprise small balls of ground or minced meat that are often mixed with other ingredients, such as breadcrumbs, starch, minced onion, eggs, and seasoning [3]. Frozen meat products are often subjected to repeated temperature cycling en route to the consumer, reducing the quality of the products [4]. In addition, repeated freeze–thaw cycles affect the sensory, physicochemical, and microbiological qualities of meat [5]. 

Various health-related ingredients, such as chia and quinoa, have been incorporated to produce value-added meat products [6,7]. Quinoa seeds are an excellent raw material for various processed foods. They are a good source of protein (12–20%), contain all essential amino acids, and do not contain gluten-type protein. They are also high in dietary fiber (10%), minerals (magnesium, phosphorus, and iron), vitamins, and other beneficial compounds, such as antioxidative and bioactive flavonoids [8,9,10,11]. Starch is the major component of quinoa seeds, comprising 53.5%–69.2% of the dry matter. Nonetheless, quinoa starch has a low glycemic index [12]. Quinoa starch has small granules (1–2 µm) and can be used to produce a creamy, smooth texture that exhibits properties similar to those of fat [13]. The amylose content of various quinoa starch is significantly lower (9.43~10.90%) than that of maize (22.58%) and potato (17.75%) starch. Quinoa starch has a high amylopectin content with good resistance to retrogradation [14]. The low amylose content and unique amylopectin structure influence the functional properties of quinoa starch [13,15]. Quinoa starch also has excellent freeze–thaw stability and, as a result, can be applied in frozen products [16,17]. Contreras-Jiménez et al. [18] reported the physicochemical properties of quinoa flour and isolated starch. They revealed that the viscosity of isolated starch had a higher value than flour, as well as its potential as a thickener in various food formulations. Quinoa and quinoa flour have also been reported to have excellent potential for use as plant-based ingredients in various meat products, including beef burgers and meatballs [7,19,20,21,22]. Quinoa can be added as a binder, filler, extender, and gelling agent in meat formulation [7]. Quinoa flour inhibits lipid oxidation during frozen storage in raw and cooked burgers [20]. Recently, Bahmanyar et al. [22] used quinoa flour in beef burgers to replace soy protein and found that the mineral content of magnesium, phosphorus, iron, and zinc was higher in the quinoa burgers than the soy protein burgers. Park et al. [23] reported the superior antioxidant activity and nutrient content of quinoa seeds cultivated in Korea.

Although quinoa starch and seeds have good potential as plant-based ingredients in meat products for health-conscious consumers, few studies have been conducted on the effect of quinoa starch and seeds on the qualities of meat products during frozen storage. In this work, we evaluated the effects of starch (quinoa and corn) and quinoa seeds on the physicochemical, textural, and sensory properties of RTC chicken meatballs during frozen storage. The effects of five freeze–thaw (F-T) cycles on the qualities of chicken meatballs were investigated for temperature fluctuation conditions during frozen storage.

## 2. Materials and Methods

### 2.1. Quinoa Starch Extraction

Quinoa (*C. quinoa* Willd.) used in this work was supplied by the Hongcheon River Farming Union (Hongcheon, Korea). Quinoa starch (QS) was extracted using the procedure proposed by Li et al. [24] with some modifications. Quinoa seeds were milled into powder for 1 min with a commercial coffee bean grinder (Crisp, CSM-309, MotorMillions Electric Industries Co., Seoul, Korea). Quinoa powder (100 g) was then stirred with 1 L of 0.2% NaOH for 30 min to remove the proteins and lipids. The residue was recovered by centrifugation (VS-15000CNF II, Vision Scientific, Daejeon, Korea) at 3000× *g* for 10 min. The removing procedure outlined above was repeated twice. The residue was subsequently suspended in distilled water and stirred overnight using a shaking incubator (HB-201SF, Hanbaek, Bucheon, Korea) to further release the protein from the starch granules. Then, the starch slurry was passed through four layers of cheesecloth and then through a 140 µm nylon mesh. The slurry was then centrifuged at 3000× *g* for 10 min, and the brown layer formed on the top of the starch layer was scraped off with a spatula and discarded. This step was repeated six times to remove the brown particles and NaOH. The resulting starch fraction was dried in an air-forced oven at 35 °C for 48 h, ground to 60 mesh powder, and sealed in an airtight plastic container until use.

### 2.2. Analysis of the Chemical Composition of Quinoa Starch 

The chemical composition of quinoa starch was determined by the AOAC methods [25]: the moisture was determined with the direct oven method (100 °C for 24 h), the ash was determined by incinerating the sample overnight at 600 °C in a muffle furnace (FX-12, Daihan Scientific. Co., Ltd., Seoul, Korea), the protein content (nitrogen × 6.25) was determined by the Kjeldahl method, and the Soxhlet extraction method with ether as the solvent was used for total fat. 

The total starch content of quinoa seeds was also determined by an enzymatic colorimetric method. The AACC-approved method 76-13.01 [26], with an assay kit from Megazyme International Ltd. (Wicklow, Ireland), was used. Quinoa seeds were ground to powder with a commercial coffee bean grinder. One hundred mg of quinoa powder was mixed with 0.2 mL of ethanol solution (80%, *v*/*v*) in a corning culture tube. A quantity of 3.0 mL of thermostable α-amylase was immediately added to a tube, boiled for 6 min, and stirred at 2 min intervals. The tube was then placed in a 50 °C water bath for 5 min, and 0.1 mL of amyloglucosidase was added to each tube. The tubes were then stirred and incubated at 50 °C for 30 min. The tube was filled with distilled water to a volume of 10 mL and then centrifuged at 1800× *g* for 10 min at room temperature (VS-550, Vision Scientific Co., Ltd., Bucheon, Korea). Next, a 1.0 mL aliquot from the supernatant was diluted with 10 mL of distilled water. Then, 0.1 mL of diluted solution was placed in a clean test tube. Glucose oxidase/peroxidase reagent (3 mL) was added to the test tube and incubated at 50 °C for 20 min. For blanks, 0.1 mL of the water was used instead of 0.1 mL of the diluted solution, while the other added reagents were the same. The absorbance of the test sample was read at 510 nm by a spectrometer-based ELISA reader (Powerwave XS, Bio-Tek Instruments, Winooski, VT, USA). The analysis was performed in duplicate. 

The amylose content of the quinoa starch was determined by the following method [27]. Quinoa starch (20 mg) was first dispersed into 10 mL of 0.5 N KOH solution with a magnetic stirring bar for 5 min until thoroughly dispersed. The dispersed sample was then transferred to 100 mL volumetric flasks and diluted with distilled water. An aliquot of the starch solution (10 mL) was pipetted into a 50 mL volumetric flask, and 5 mL of 0.l N HCl was added, followed by 0.5 mL of the iodine reagent. The volume was diluted to 50 mL with distilled water and then left for 5 min at room temperature. Then, the absorbance of the blue color was measured using a spectrometer-based ELISA reader at 620 nm. The amylopectin content was calculated by subtracting the amylose content from the total starch content.

### 2.3. Preparation of Chicken Meatballs with Starch (Corn and Quinoa) and Quinoa Seeds 

Chicken breast meat and corn starch were purchased from a local store in Seoul, Korea. Quinoa seeds were supplied by the Hongchun-river quinoa farming union (Hongchun, Korea). Quinoa seeds were washed and soaked in distilled water for 3 h to remove the bitter taste. The ground chicken meat was prepared by blending chicken breast meat using a blender (HR-2870, Philips, Seoul, Korea). According to the formulations of each sample, 10 g of quinoa seeds and 2.5 g of starch (corn or quinoa) were directly added to 100 g of the ground chicken meat. There were five treatment groups: CS—corn starch was added; QS—quinoa starch was added; Q—quinoa seeds were added; CS-Q—corn starch and quinoa seeds were added; and QS-Q—quinoa starch and quinoa seeds were added (Table 1).

The ground chicken meat was then mixed with 9.5% onion, 5% water, 2.5% breadcrumbs, 2% garlic, 0.3% salt, 0.2% pepper, and 0.05% ginger for 1 min to make the batter, which was shaped into 15 g balls (2.5 cm in diameter and 1.5 cm in height). Thirty-six chicken meatballs were prepared, and 6 samples for each treatment were used for physicochemical property analysis. Additionally, 40 chicken meatballs were prepared for sensory evaluation. Chicken meatballs were steamed for 30 min using an electric steamer (FineArt, Incheon, Korea) to keep the internal temperature at 63 ± 2 °C. A waterproof pocket digital thermometer (Daihan Scientific Co., Wonju, Korea) is immediately inserted into chicken meatballs to check the internal temperature. After heating, chicken meatballs were cooled to room temperature for 2 h, packed into polyethylene bags (Tobbigs, Hwaseong, Korea), and then stored at −18 °C. The samples were subjected to 5 freeze–thaw cycles (F-T cycles) for accelerated frozen storage and (1 F-T cycle; frozen at −18 °C for seven days and thawed at 4 °C for one day) to measure the effects of freeze–thaw cycles on the quality characteristics of chicken meatballs. Six chicken meatballs for each treatment were analyzed after each F-T cycle. 

### 2.4. Water-Holding Capacity (WHC)

Cooking, drip, and reheating losses were measured as an index for water-holding capacity using an electronic balance (Scaltech SBA 51, Heiligenstadt, Germany) with an accuracy of 0.01 g. The weight of chicken meatballs was measured before and after cooking (steaming for 30 min) to determine the cooking loss. For drip loss, the weight of the chicken meatball was measured before and after each F-T cycle. Thawed, cooked chicken meatballs were reheated in a microwave for 1 min to reach the internal temperature of 98 °C to determine reheating loss. A waterproof pocket digital thermometer was immediately inserted into the chicken meatballs to check the internal temperature after microwave cooking. Percentages of cooking, drip, and reheating losses were determined by calculating the weight differences of the samples before and after cooking, freezing, and reheating, as shown in the equation:

Cooking loss, drip loss, and reheating loss (%) = [(W_1_ − W_2_)/W_1_] × 100 (1)

where W_1_ represents sample weight (g) before cooking, freezing, and reheating, and W_2_ represents the sample weight (g) after cooking, freezing, and reheating. Three samples were analyzed for each treatment.

### 2.5. Determination of Total Volatile Basic Nitrogen (TVB-N) 

TVB-N content was determined using the micro-diffusion method [28]. A quantity of 5 g of chicken quinoa meatballs in each F-T cycle was mixed with 25 mL of distilled water and adequately homogenized. Then, the meatballs were left for 30 min at an ambient temperature and filtered through filter paper (Whatman no. 1). A sealing agent (glycerin) was applied to the edge of the outer ring of Conway’s unit. A quantity of 1 mL of the filtered solution was pipetted into the outer ring of each unit. A quantity of 1 mL of 0.01 N HCl was pipetted into the inner ring of each unit, while 1 mL of saturated K_2_CO_3_ solution was carefully pipetted into the outer ring, and the units were immediately covered and closed with a clip. The solutions in the units were then gently mixed to prevent any solution from mixing from one ring to the other. The units were placed at 25 °C for 60 min. Then, 1 drop of the indicator solution (0.2 g of methyl red and 0.1 g of methylene blue in 300 mL of ethanol) was added to the inner ring solution and titrated with 0.01 N NaOH using a burette until the purple color turned green. An average titrate volume of NaOH was found from the results of three titrations for each chicken meatball. A blank test was also performed using 1 mL of distilled water instead of the sample solution. The TVB-N values were calculated using the following equation:

TVB-N (mg %) = 0.14 × ((b − a) × f)/w × 100 × d(2)

- a: total volume of NaOH used to titrate for sample solution (mL)
- b: total volume of NaOH used to titrate for distilled water (mL)
- f: factor of 0.01 N NaOH, w: sample weight (g), d: dilution rate

### 2.6. Determination of Thiobarbituric Acid Reactive Substances (TBARS)

A 2-thiobarbituric acid reactive substances (TBARS) assay was performed using a modified method [29] to determine lipid oxidation, based on spectrophotometric analysis of the pink complex formed after the reaction of malondialdehyde (MDA) with 2-thiobarbituric acid (Sigma-Aldrich Co., St. Louis, MO, USA). 

Five grams of chicken meatball after each F-T cycle was weighed into a conical tube (50 mL) with 15 mL of distilled water and homogenized with 50 μL of 7.2% butylated hydroxytoluene in ethanol for 30 s to stop the oxidation reaction, and then placed in a darkroom for 15 min. The homogenate was centrifuged at 2259× *g* for 15 min (VS-550, Vision Scientific Co., Ltd., Bucheon, Korea) and filtered using No.1 filter paper (ADVANTEC, Bunkyo, Tokyo, Japan). One mL of the filtered solution was placed in a conical tube (15 mL), and 2 mL of the TBA/TCA (thiobarbituric acid/trichloroacetic acid) solution was added. The mixture was vortexed, heated at 100 °C for 15 min, and refrigerated for 10 min. The mixture was then centrifuged at 737× *g* for 15 min, and the absorbance of the supernatant was measured using a spectrometer-based ELISA reader at 532 nm (Powerwave XS, BioTEk Instruments Inc., Winooski, VT, USA). A blank test was also performed using 1 mL of distilled water instead of the sample solution. The TBARS value (mg malondialdehyde per kg of the sample) was calculated using the following equation:

TBARS value = (a − b) × 5.88 mg malondialdehyde/kg of sample(3)

where a represents the absorbance of the sample, b represents the absorbance of the blank, and 5.88 represents the slope of the malondialdehyde standard curve.

### 2.7. Determination of DPPH Free Radical Scavenging Activity

The antioxidant activity of the sample was determined using a modified 2, 2-diphenyl-1- picrylhydrazyl (DPPH) free radical scavenging assay [30]. A quantity of 5 g of chicken meatball after each F-T cycle was homogenized (BagMixer^®^ 400W, Interscience, St. Nom la Bretêche, Île-de-France, France) with 45 mL of 0.1 M sodium phosphate buffer (pH 7.0) for 2 min. Then, the homogenate was filtered using No.1 filter paper (ADVANTEC, Bunkyo, Tokyo, Japan) to remove impurities and then centrifuged at 12,000× *g* for 30 min. A volume of 1 mL of 0.2 mM DPPH dissolved in methanol (Sigma-Aldrich Co., St. Louis, MO, USA) was added to 200 μL of the supernatant and 800 μL of distilled water in 15 mL of the conical tube. The mixture was vortexed and placed in a darkroom for 30 min. The conical tube containing 1 mL of methanol and 1 mL of 0.2 mM DPPH dissolved in methanol was used as the control, whereas 2 mL of methanol alone was used as a blank. The absorbance of the sample solution was measured using a spectrometer-based ELISA reader at 517 nm. The scavenging activity of the sample against the DPPH radical was calculated using the following equation:

(4) $$\%\;\mathrm{inhibition}\;\mathrm{of}\;\mathrm{DPPH}=\frac{\left[\left(\mathrm{Abs}\;\mathrm{control}-\mathrm{Abs}\;\mathrm{blank}\right)-\left(\mathrm{Abs}\;\mathrm{sample}-\mathrm{Abs}\;\mathrm{blank}\right)\right]}{\mathrm{Abs}\;\mathrm{control}}\;\times \;100$$

### 2.8. Instrumental Color Analysis

The colorimetric parameters, including the L*, a*, and b* values, of the chicken meatballs were measured using a colorimeter (CR-400, Minolta, Osaka, Japan: 2° closely matches CIE 1931 standard observer and pulsed xenon lamp for light source) after each F-T cycle to analyze the effect of the freeze–thaw process on the color of chicken meatballs. The colorimeter was standardized against a white standardization plate (Y = 93.5, x = 0.3114, and y = 0.3190) before each measurement. All measurements were performed on the surface of chicken meatballs in triplicate. The color was described with the following coordinates: lightness (L*), chroma (C*), and hue (h*). The chroma and hue were calculated by √a*^2^ + b*^2^ and tan^−1^ (b*/a*), respectively. The effect of 5 F-T cycles on the L*a*b* color difference of chicken meatballs was also calculated using the following equation:

ΔE = √(ΔL)^2^ + (Δa)^2^ + (Δb)^2^. (5)

### 2.9. Texture Profile Analysis (TPA) of Chicken Meatballs

After each freeze–thaw cycle, three whole chicken meatballs (2.5 cm in diameter and 1.5 cm in height) were reheated, cooled to room temperature, and then subjected to texture profile analysis using a texture analyzer (CT3-10K, AMETEK Brookfield, Middleboro, MA, USA). The conditions of the texture analyzer were as follows: pre-test speed, 2.0 mm/s; post-test speed, 5.0 mm/s; sample rate, 10 points/sec; test target type, 90% deformation; trigger load, 10 g; test speed, 0.5 mm/s; probe, TA4/1000 cylinder (38.1 mm D, 20 mm L). The calculation of the TPA values was obtained by graphing a curve using force and time plots. Values for hardness, adhesiveness, springiness, cohesiveness, gumminess, and chewiness were measured.

### 2.10. Acceptance Test of Chicken Meatballs

The cooked chicken meatballs were frozen at −20 °C before sensory evaluation. Each precooked meatball was reheated in a microwave oven at high power (700 W) for 1 min to reach an internal temperature of 98 °C. The cooked chicken meatballs were cooled to room temperature and served to panelists in a random order. Water was provided to clear the palate of residual flavors between the samples. Thirty-five semi-trained panelists measured the acceptability of the sensory properties, including the appearance, color, odor, flavor, taste, texture, and overall acceptability, using a 9-point hedonic scale. The values on the scale indicated the following range of reactions: 1: dislike extremely to 9: like extremely.

### 2.11. Statistical Analyses

All the experiments were performed in triplicate. All data were expressed as mean values and standard deviation and were analyzed with SAS software version 9.3 (SAS Institute, Cary, NC, USA). One-way ANOVA was performed for the analysis of variance, and the significant differences among treatments were determined with Duncan’s multiple range test at *p* < 0.05.

## 3. Results and Discussion

### 3.1. Chemical Composition of Quinoa Starch and Water-Holding Capacity 

The chemical composition of starch isolated from quinoa cultivated in Korea was analyzed. The starch contained 10.51% moisture, 0.7% crude ash, 0.55% crude protein, 1.88% crude fat, and 89.86% total starch. These results are consistent with those reported by Steffolani et al. [12], who studied the physicochemical and functional properties of starch isolated from various quinoas. In this work, the isolated quinoa starch, which was cultivated in Korea, contained 12.52% amylose and 87.48% amylopectin, which generally agrees with the previous studies [15,31]. 

The effects of quinoa seeds and starch on the cooking, drip, and reheating losses of chicken meatballs were measured after cooking, F-T cycles, and reheating by a microwave oven, respectively, to measure the water-holding capacity of chicken meatballs (Table 2). Overall, the cooking losses of chicken meatballs containing quinoa seeds (Q) was about 1.8-fold higher than those of chicken meatballs with starch, indicating an increase in the water-holding capacity due to the addition of starch in the meatballs. However, the kind of starch (CS or QS) did not significantly affect cooking loss. In particular, the combination of quinoa seeds with starch resulted in the lowest cooking loss among the chicken meatballs studied. Cooking loss due to non-meat ingredients or other factors is the most critical factor for the meat industry in predicting the behavior of products during cooking [32]. Meatballs tend to shrink during the cooking process due to the denaturation of meat proteins, and the loss of water and fat also contributes to the shrinking process [33]. 

The drip loss of chicken meat significantly increased by 61% after six F-T cycles [34]. No difference in drip loss was found following the addition of starch to the chicken meatballs until the meatballs were subjected to four F-T cycles. After five F-T cycles, the meatballs with Q showed the highest drip loss (1.96%), followed by the meatballs with CS (1.53%) and the meatballs with QS (1.49%). Although the cooking and drip losses of chicken meatballs with QS did not significantly differ from those of chicken meatballs with CS in the present study, the water-holding capacity of QS was slightly better than that of CS. Adding starch reduced the drip and reheating losses of chicken meatballs, suggesting that starch is essential to prevent drip losses of chicken meatballs during frozen storage and to retain product moisture during reheating. Only chicken meatballs containing Q did not prevent drip and reheating losses during F-T cycles in this work. This result indicates that the addition of starch significantly affected the water-holding capacity of chicken meatballs. Again, the reheating loss of chicken meatballs with QS was also less than those with CS after three F-T cycles. Prestes et al. [35] also reported that using corn starch in low-fat chicken mortadella resulted in a higher value of reheating loss than that of cassava starch, although the difference did not reach statistical significance. Regular CS has around 28.7% amylose [36], whereas the quinoa starch used in this work has 12.52% amylose. Starches with a high content of amylose present a high value of gel strength due to the long linear chains of polymers linked by hydrogen bonds to the gel matrix. Amylose molecules tend to reassociate with concomitant liquid release from the gel during storage, thus influencing the liquid losses of meat products [37]. In the present study, we found that QS has an excellent capacity to prevent water loss during cooking, thawing, and reheating.

### 3.2. Effect of Quinoa Seeds and Starch on Total Volatile Basic Nitrogen (TVB-N), Thiobarbituric Acid Reactive Substances (TBARS), and DPPH Free Radical Scavenging Activity

The total volatile basic nitrogen (TVB-N) content in meat products is an important chemical index used to evaluate the products’ freshness [38]. As the length of the storage period increases, the main ingredients, such as proteins, fats, and carbohydrates, are decomposed by enzymes and microbes into smaller toxic molecular components, such as ammonia, trimethylamine (TMA), and dimethylamine (DMA). The TVB-N compounds in meat products increase relative to spoilage [39]. The TVB-N values of the chicken meatballs in this study significantly increased during five F-T cycles. The average TVB-N value of chicken meatballs increased from 10.39 mg% at zero F-T cycles to 14.56 mg% at five F-T cycles (*p* < 0.05). However, the TVB-N level did not reach 20 mg%, which is the limit for fresh meat products in Korea [23], indicating that the TVB-N value of chicken meatballs was not the main quality index for frozen chicken meatballs. These results are generally consistent with Patsias et al. [40], who reported TVB-N values in the range of 25 mg% for freeze-chilled chicken breast fillets after 15 days. Moreover, the addition of CS, QS, or Q did not significantly affect the TVB-N value of chicken meatballs during five F-T cycles (*p* > 0.05).

Lipid oxidation is a critical factor for the quality of meat products. TBARS analysis was used to evaluate the lipid oxidation of chicken meatballs during five F-T cycles, for which malondialdehyde (the secondary product of lipid oxidation) in the sample was measured for the TBARS value. Overall, the TBARS values of chicken meatballs increased (*p* < 0.05) with increased F-T cycles. Chicken meatballs with or without starch showed no distinct change in TBARS values during each F-T cycle. At five F-T cycles, the TBARS values of CS, QS, Q, CS-Q, and QS-Q were 0.90, 0.84, 0.85, 0.81, and 0.79 mg in the malonaldehyde (MDA)/kg sample, respectively. Chicken meatballs containing QS-Q had significantly less TBARS values than those with CS only (Table 3), indicating that QS-Q prevented the production of malondialdehyde in chicken meatballs during F-T cycles. Meat products are well preserved from oxidative changes when they have less than 3 mg MDA/kg sample [41]. All chicken meatballs showed TBARS values below 3 mg MDA/kg after five F-T cycles in the present study, indicating that the samples underwent a progressive but not rapid increase in lipid oxidation, even after five F-T cycles. These results agree with the findings of Song et al. [42], who reported that the TBARS value in chicken meatballs increased during frozen storage at −20 °C for 12 weeks but did not reach the level of 3 mg MDA/kg. In this work, chicken meatballs containing CS-Q or QS-Q showed significantly lower TBARS values after five F-T cycles compared with those with CS, QS, or Q only. 

Overall, the DPPH values in chicken meatballs with quinoa seeds were higher than those in chicken meatballs without Q (*p* < 0.05), indicating that the addition of Q improved the antioxidant effects of chicken meatballs. These results are in agreement with Alvarez-Jubete et al. [43], who observed that the addition of quinoa seeds in bread was found to increase total antioxidant activity by 19% compared with wheat flour. In previous studies [44,45], the effect of the heating process on the total antioxidant activities of quinoa was determined. Cooking does not influence any significant changes in the total phenolic acid content, total flavonoid content, and antioxidant activity of red quinoa seeds [44]. The overall antioxidant activities of quinoa seeds are increased in washed and cooked samples, particularly when prepared with pressure [45]. The DPPH values of all chicken meatballs significantly decreased as the number of F-T cycles increased (Table 3). The addition of both CS and QS did not significantly affect the antioxidant activities of chicken meatballs. However, the DPPH value of chicken meatballs with CS or QS sharply decreased at four F-T cycles and was not detected after five F-T cycles. These results indicate that repeated F-T cycles decreased the antioxidative activity of chicken meatballs, but the reduction was minimized with the addition of Q in chicken meatballs.

### 3.3. Effect of Quinoa Starch and Seeds on the Textural Properties of Chicken Meatballs

**Figure 1:**
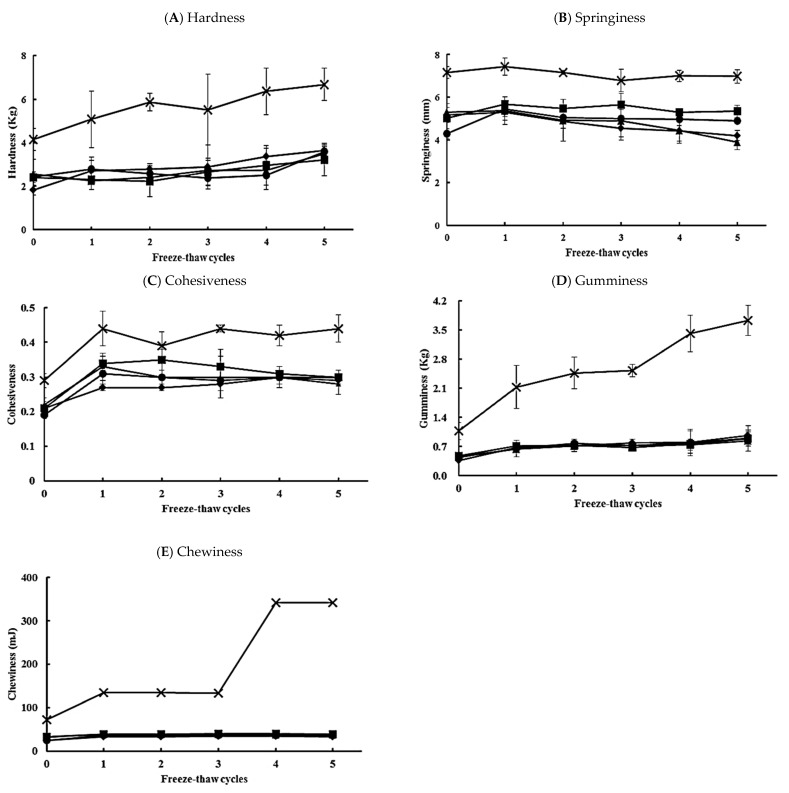
Texture profile analysis (TPA) of chicken meatballs during five freeze-thaw cycles (F-T cycles). (**A**) Hardness. (**B**) Springiness. (**C**) Cohesiveness. (**D**) Gumminess. (**E**) Chewiness. CS, corn starch (▲); QS, quinoa starch (■); Q, quinoa seed (**×**); CS-Q, corn starch and quinoa seed (●); QS-Q, quinoa starch and quinoa seed (♦).

The texture of chicken meatballs with CS, QS, Q, CS-Q, or QS-Q was evaluated (Figure 1). Multiple freeze–thaw cycles increased (*p* < 0.05) all the textural parameters of the chicken meatballs, with the exception of springiness. After one F-T cycle, the cohesiveness, gumminess, and chewiness of chicken meatballs significantly increased, indicating that frozen storage affected the texture of chicken meatballs. In particular, the chewiness (Figure 1E) of chicken meatballs containing Q between zero and one cycle doubled from 71.17 to 133.97 mJ. In processed poultry products, starches are commonly used as bulking agents to improve texture characteristics, as thickeners, and for gelling and water retention [37]. The values of hardness, gumminess, and chewiness of meatballs containing Q were twice as high as those of other chicken meatballs with starch (CS, QS, CS-Q, QS-Q). However, hardness, cohesiveness, and gumminess were not significantly affected (*p* > 0.05) by the kind of starch (CS or QS). After five F-T cycles, the springiness (Figure 1B) and chewiness (Figure 1E) of chicken meatballs with CS or CS-Q were higher than those of meatballs with QS or QS-Q. The types and amounts of extenders, such as starch, play a decisive role in the resulting texture of meatballs [33]. In addition, variation in the amount of amylose affects the texture of starch-containing foods. Starches containing a higher amount of amylose tend to form a gel, whereas starches containing a higher level of amylopectin do not form a gel and tend to provide a gummy texture [46]. Regular CS has around 28.7% amylose [36], whereas the isolated QS in the present study has 12.52% amylose. The hardness values of gel with CS and QS were 27.28 g and 20.8 g, respectively [47]. However, no significant differences in the textural properties of chicken meatballs were observed between CS and QS in this work. The textural properties of quinoa flour have been investigated in various meat products, but few papers have looked at the textural properties of QS. More studies should evaluate the possibility of applying quinoa starch in various food products in the future. The addition of starch decreased the chewiness of chicken meatballs. The range of chewiness of the chicken meatballs with CS or QS and those with CS-Q or QS-Q was 32.00–40.07 mJ and 24.43–36.87 mJ, respectively (Figure 1E). However, the chewiness value of chicken meatballs containing only Q was significantly higher than those with starch. These results indicate that QS may contribute to the meaty texture of various meatless burgers.

### 3.4. Effect of Quinoa Seeds and Starch on the Color of Chicken Meatballs during F-T Cycles 

As the F-T cycle increased, the L* values (lightness) of the chicken meatballs also increased (Table 4), indicating that the color of the chicken meatballs became lighter due to freezer burn during repeated F-T cycles. The addition of starch and quinoa seeds resulted in higher and lower L* values, respectively, indicating that the addition of Q in chicken meatballs decreased the lightness of the surface color of meatballs due to the brown color of QS. The chicken meatballs with CS also showed higher L* values than those with QS without significant difference. Significantly higher C* values were observed in chicken meatballs with QS than those with CS, indicating that a stronger color of chicken meatballs with QS can be obtained. The highest C* values of chicken meatballs with QS-Q were observed among chicken meatballs during five F-T cycles. Higher b* values (yellowness) were observed in chicken meatballs containing Q (data not shown) due to the brownish color of quinoa seeds, which resulted in a higher C* value. Although no changes in redness (a*) were observed during the F-T process (data not shown), the repeated F-T cycle decreased the C* values, indicating that the color of chicken meatballs became a paler gray. The addition of CS decreased h* values significantly (*p* < 0.05), and thus the lowest h* value was observed in chicken meatballs with CS-Q, followed by QS-Q during five F-T cycles. 

According to Ikhlas et al. [33], different flours resulted in different quail meatballs. Compared with cassava and potato flour, quail meatballs using corn flour produced higher L* values and lower a* and b* values. After five F-T cycles, a minor L*a*b* color difference was observed in chicken meatballs containing QS (0.39), followed by CS (2.21), Q (2.62), QS-Q (3.28), and CS-Q (3.97). This result indicates that the addition of QS also minimized the color change of chicken meatballs during five F-T cycles. 

### 3.5. Effect of Quinoa Seeds and Starch on the Sensory Score

After frozen storage, the sensory qualities of reheated chicken meatballs were evaluated in terms of appearance, color, flavor, taste, texture, and overall acceptability using a nine-point hedonic scale (Table 5). The chicken meatballs with Q, CS-Q, and QS-Q had lower (*p* < 0.05) appearance scores than those with CS and QS. The lowest taste, texture, and overall acceptability scores were recorded in chicken meatballs with Q only, indicating that the addition of starch increased the sensory properties of chicken meatballs. However, the kind of starch did not significantly affect the sensory properties of chicken meatballs. No differences in color and flavor scores were found among all tested chicken meatballs (*p* > 0.05). Although the chicken meatballs with Q had a lower appearance score compared with other chicken meatballs, the highest overall acceptability score was observed in chicken meatballs combining QS-Q. Similarly, Stikic et al. [48] reported that bread supplemented with 15% of quinoa seeds had excellent sensory characteristics. Similarly, dark chocolate with quinoa seeds in the proportions of 12%, 16%, and 20% was approved by 92% of the sensory panel with an acceptance index above 70% [49].

In conclusion, adding QS and Q affects the physicochemical, textural, and sensory properties of frozen chicken meatballs. The addition of QS produced fewer cooking, drip, and reheating losses. No significant differences in the TVB-N and TBARS values among chicken meatballs with different ingredients were observed after five F-T cycles, indicating that the qualities of chicken meatballs related to protein deterioration and fat oxidation were not significantly affected during frozen storage. The antioxidant activity in chicken meatballs with Q was improved considerably. The addition of CS or QS prevented a color and texture change of chicken meatballs during F-T cycles. Overall, the addition of QS-Q improved the water-holding capacity, antioxidant activity, and textural properties of chicken meatballs during repeated F-T cycles. Both QS and Q can be used as functional meat products in the premium frozen market. In particular, Q may help provide a meaty texture in various meatless products. The physicochemical properties of QS and Q provide functional properties that are suitable for novel uses. Since few studies have examined the textural properties of QS, more studies involving sensory evaluation should be conducted to evaluate its application potential in various food products.

## Figures and Tables

**Table 1:** Formulations of the chicken meatballs with quinoa seeds and starch.

Ingredient (g)	CS	QS	Q	CS-Q	QS-Q
Chicken breast meat	77.95	77.95	70.45	67.95	67.95
Quinoa seeds	0	0	10	10	10
Corn starch	2.5	-	-	2.5	-
quinoa starch	-	2.5	-	-	2.5

CS, corn starch; QS, quinoa starch; Q, quinoa seeds; CS-Q, corn starch and quinoa seeds; QS-Q, quinoa starch and quinoa seeds.

**Table 2:** Cooking loss, drip loss, and reheating loss of chicken meatballs during five freeze-thaw cycles (F-T cycles).

	F-T Cycles	CS	QS	Q	CS-Q	QS-Q
Cooking loss (%)	0	15.30 ± 0.35 ^B^	15.35 ± 0.59 ^B^	25.87 ± 0.73 ^A^	14.03 ± 0.77 ^C^	13.86 ± 0.43 ^C^
Drip loss (%)	1	1.15 ± 0.03 ^Abc^	1.10 ± 0.06 ^Acd^	0.80 ± 0.26 ^Bc^	0.95 ± 0.06 ^ABc^	0.70 ± 0.18 ^Bc^
	2	1.10 ± 0.11 ^Ac^	1.03 ± 0.04 ^Ad^	1.19 ± 0.03 ^Ab^	1.12 ± 0.12 ^Abc^	1.03 ± 0.13 ^Ab^
	3	1.27 ± 0.17 ^Abc^	1.24 ± 0.08 ^Abc^	1.37 ± 0.19 ^Ab^	1.21 ± 0.21 ^Aabc^	1.14 ± 0.10 ^Aab^
	4	1.37 ± 0.01 ^Aab^	1.29 ± 0.15 ^Ab^	1.45 ± 0.16 ^Ab^	1.33 ± 0.14 ^Aab^	1.28 ± 0.13 ^Aab^
	5	1.53 ± 0.17 ^Ba^	1.49 ± 0.11 ^Ba^	1.96 ± 0.27 ^Aa^	1.48 ± 0.27 ^Ba^	1.42 ± 0.23 ^Ba^
Reheating loss (%)	1	21.71 ± 0.35 ^Ce^	21.34 ± 0.41 ^Cc^	26.30 ± 0.51 ^Ae^	23.45 ± 0.53 ^Bc^	23.80 ± 0.58 ^Bc^
	2	22.48 ± 0.59 ^Cd^	23.22 ± 0.62 ^Bb^	27.29 ± 0.88 ^Ad^	23.44 ± 0.26 ^BCc^	24.07 ± 0.06 ^Bbc^
	3	24.80 ± 0.15 ^Bc^	23.72 ± 1.09 ^Cb^	28.61 ± 0.35 ^Ac^	23.39 ± 0.21 ^Cc^	24.41 ± 0.41 ^BCbc^
	4	25.65 ± 0.37 ^Bb^	24.51 ± 0.86 ^Cb^	30.14 ± 0.35 ^Ab^	24.66 ± 0.28 ^Cb^	24.72 ± 0.29 ^Cb^
	5	28.75 ± 0.22 ^Ba^	28.48 ± 0.36 ^Ba^	31.41 ± 0.25 ^Aa^	28.22 ± 0.26 ^Ba^	28.71 ± 0.32 ^Ba^

CS, corn starch; QS, quinoa starch; Q, quinoa seeds; CS-Q, corn starch, and quinoa seeds; QS-Q, quinoa starch, and quinoa seeds. Values are expressed as mean (*n* = 3) and standard deviation. ^a–e^ Mean values with different letters in the same column within the same row are significantly different according to Duncan’s multiple range test (*p* < 0.05), compared among F-T cycles. ^A–C^ Mean values with different letters in the same row are significantly different according to Duncan’s multiple range test at *p* < 0.05, compared among chicken meatballs.

**Table 3:** Total volatile basic nitrogen (TVB-N), thiobarbituric acid reactive substances (TBARS) value, and diphenyl-1- picrylhydrazyl (DPPH) value of the chicken meatballs during five F-T cycles.

	F-T Cycles	CS	QS	Q	CS-Q	QS-Q
TVB-N (mg %)	0	10.10 ± 0.69 ^d^	10.73 ± 0.81 ^b^	0.94 ± 2.28 ^b^	10.52 ± 0.84 ^c^	9.68 ± 0.49 ^d^
	1	12.41 ± 0.81 ^c^	12.94 ± 0.40 ^a^	13.05 ± 1.09 ^a^	13.05 ± 0.77 ^b^	12.20 ± 1.09 ^c^
	2	13.05 ± 0.60 ^bc^	13.15 ± 0.40 ^a^	13.68 ± 1.26 ^a^	13.68 ± 1.06 ^ab^	13.26 ± 0.42 ^bc^
	3	13.68 ± 0.54 ^ab^	13.68 ± 1.26 ^a^	13.89 ± 0.49 ^a^	13.36 ± 0.72 ^ab^	13.57 ± 0.99 ^bc^
	4	13.99 ± 0.63 ^ab^	13.78 ± 0.63 ^a^	14.10 ± 0.81 ^a^	13.05 ± 0.49 ^b^	15.26 ± 1.83 ^a^
	5	14.73 ± 0.60 ^a^	14.39 ± 2.90 ^a^	14.52 ± 0.42 ^a^	14.52 ± 0.42 ^a^	14.62 ± 0.40 ^ab^
TBARS (mg malonaldehyde/kg of sample)	0	0.11 ± 0.01 ^Af^	0.09 ± 0.01 ^Bf^	0.11 ± 0.03 ^Ad^	0.11 ± 0.01 ^Ad^	0.12 ± 0.02 ^Ad^
	1	0.30 ± 0.01 ^Be^	0.32 ± 0.01 ^Be^	0.31 ± 0.03 ^Bc^	0.36 ± 0.02 ^Ac^	0.31 ± 0.01 ^Bc^
	2	0.38 ± 0.08 ^Ad^	0.39 ± 0.04 ^Ad^	0.38 ± 0.04 ^Ac^	0.39 ± 0.05 ^Ac^	0.33 ± 0.06 ^Ac^
	3	0.67 ± 0.07 ^Ac^	0.67 ± 0.04 ^Ac^	0.65 ± 0.09 ^Ab^	0.70 ± 0.14 ^Ab^	0.69 ± 0.08 ^Ab^
	4	0.74 ± 0.06 ^ABb^	0.73 ± 0.04 ^ABb^	0.69 ± 0.06 ^Bb^	0.78 ± 0.08 ^Aab^	0.72 ± 0.05 ^ABb^
	5	0.90 ± 0.03 ^Aa^	0.84 ± 0.03 ^ABa^	0.85 ± 0.10 ^ABa^	0.81 ± 0.07 ^Ba^	0.79 ± 0.04 ^Ba^
DPPH free radical-scavenging activity (%)	0	20.28 ± 0.58 ^Ba^	20.73 ± 0.29 ^Ba^	34.51 ± 0.63 ^Aa^	34.84 ± 0.34 ^Aa^	34.41 ± 0.21 ^Aa^
	1	18.42 ± 1.69 ^Cb^	17.26 ± 0.97 ^Cb^	33.33 ± 1.03 ^Bb^	33.60 ± 0.67 ^ABb^	34.82 ± 0.47 ^Aa^
	2	17.72 ± 0.48 ^Cb^	18.16 ± 0.84 ^Cb^	25.25 ± 0.54 ^Ac^	25.23 ± 0.97 ^Ac^	22.23 ± 0.72 ^Bb^
	3	15.95 ± 1.21 ^Cc^	15.95 ± 1.21 ^Cc^	22.08 ± 0.80 ^Ad^	20.43 ± 1.48 ^Bd^	19.36 ± 1.03 ^Bc^
	4	5.42 ± 0.80 ^Cd^	8.06 ± 1.73 ^Bd^	20.85 ± 0.34 ^Ae^	20.31 ± 1.37 ^Ad^	19.24 ± 1.84 ^Ac^
	5	ND	ND	12.22 ± 1.78 ^Af^	11.04 ± 0.73 ^Ae^	10.87 ± 0.65 ^Ad^

CS, corn starch; QS, quinoa starch; Q, quinoa seeds; CS-Q, corn starch, and quinoa seeds; QS-Q, quinoa starch, and quinoa seeds. ND: Not detected, values are expressed as mean (*n* = 3) and standard deviation. ^a–f^ Mean values with different letters in the same column within the same row are significantly different according to Duncan’s multiple range test (*p* < 0.05), compared among F-T cycles. ^A–C^ Mean values with different letters in the same row are significantly different according to Duncan’s multiple range test at *p* < 0.05, compared among chicken meatballs.

**Table 4:** Instrumental color (L*, a*, and b*) of chicken meatballs during five F-T cycles.

	F-T Cycles	CS	QS	Q	CS-Q	QS-Q
L* (lightness)	0	79.53 ± 0.22 ^Ab^	79.47 ± 0.13 ^Aab^	73.82 ± 0.09 ^Bd^	74.00 ± 0.41 ^Bd^	73.89 ± 0.46 ^Bd^
	1	79.50 ± 0.43 ^Ab^	79.55 ± 0.39 ^Aab^	75.15 ± 0.61 ^Bc^	75.01 ± 0.53 ^Bc^	74.59 ± 0.38 ^Bc^
	2	80.08 ± 1.10 ^Ab^	79.63 ± 0.28 ^Aa^	75.72 ± 0.03 ^Bb^	75.92 ± 0.56 ^Bb^	75.52 ± 0.41 ^Bb^
	3	81.27 ± 0.19 ^Aa^	79.93 ± 0.06 ^Ba^	75.87 ± 0.18 ^Cb^	76.35 ± 0.58 ^Cb^	75.20 ± 0.10 ^Db^
	4	81.24 ± 0.06 ^Aa^	79.15 ± 0.20 ^Bb^	76.82 ± 0.07 ^Da^	77.44 ± 0.28 ^Ca^	75.28 ± 0.12 ^Eb^
	5	81.67 ± 0.15 ^Aa^	79.54 ± 0.25 ^Bab^	76.20 ± 0.04 ^Eb^	77.67 ± 0.19 ^Ca^	76.50 ± 0.06 ^Da^
C* (chroma/ intensity)	0	14.34 ± 0.27 ^Ca^	15.09 ± 0.12 ^BCa^	15.87 ± 0.14 ^ABa^	15.61 ± 0.34 ^ABa^	16.66 ± 1.26 ^Aa^
	1	14.13 ± 0.05 ^Da^	15.01 ± 0.14 ^BCab^	15.50 ± 0.30 ^ABb^	14.81 ± 0.28 ^Cc^	15.67 ± 0.58 ^Aabc^
	2	13.74 ± 0.04 ^Cb^	14.99 ± 0.31 ^Bab^	15.38 ± 0.14 ^Ab^	14.81 ± 0.06 ^Bc^	15.34 ± 0.05 ^Abc^
	3	13.52 ± 0.11 ^Cb^	15.17 ± 0.07 ^Ba^	15.53 ± 0.22 ^ABb^	15.18 ± 0.04 ^Bb^	15.74 ± 0.47 ^Aab^
	4	13.64 ± 0.18 ^Db^	14.95 ± 0.06 ^Bab^	15.48 ± 0.01 ^Ab^	14.62 ± 0.09 ^Cc^	15.08 ± 0.13 ^Bbc^
	5	13.80 ± 0.17 ^Bb^	14.72 ± 0.06 ^Ab^	14.78 ± 0.05 ^Ac^	14.49 ± 0.16 ^Ac^	14.71 ± 0.18 ^Ac^
h* (hue)	0	84.96 ± 0.71 ^Ba^	85.74 ± 0.19 ^Aa^	85.88 ± 0.05 ^Aa^	83.71 ± 0.20 ^Ca^	85.08 ± 0.04 ^Bb^
	1	85.17 ± 0.96 ^Aa^	85.99 ± 0.08 ^Aa^	85.56 ± 0.58 ^Aa^	84.00± 0.51 ^Ba^	85.83 ± 0.54 ^Aa^
	2	85.74 ± 0.11 ^Aa^	86.02 ± 0.38 ^Aa^	85.56 ± 0.59 ^Aa^	83.81 ± 0.05 ^Ba^	84.31 ± 0.21 ^Bc^
	3	85.72 ± 0.26 ^Aa^	85.92 ± 0.17 ^Aa^	85.53 ± 0.01 ^Aa^	83.34 ± 0.63 ^Ca^	84.32 ± 0.23 ^Bc^
	4	85.67 ± 0.03 ^Aa^	86.05 ± 0.45 ^Aa^	85.52 ± 0.18 ^Aa^	83.32 ± 0.83 ^Ba^	83.99 ± 0.10 ^Bcd^
	5	85.60 ± 0.07 ^Aa^	86.11 ± 0.38 ^Aa^	85.38 ± 0.84 ^Aa^	83.30 ± 0.08 ^Ba^	83.64 ± 0.43 ^Bd^
ΔE*		2.21	0.39	2.62	3.84	3.28

CS, corn starch added; QS, quinoa starch; Q, quinoa seeds; CS-Q, corn starch and quinoa seeds; QS-Q, quinoa starch and quinoa seeds values are expressed as mean (*n* = 3) and standard deviation. ΔE* = √ΔL^2^ + Δa^2^ + Δb^2^: calculated difference between zero F-T cycles and five F-T cycles. ^a–d^ Mean values with different letters in the same column within the same row are significantly different according to Duncan’s multiple range test (*p* < 0.05), compared among F-T cycles. ^A–E^ Mean values with different letters in the same row are significantly different according to Duncan’s multiple range test at *p* < 0.05, compared among chicken meatballs.

**Table 5:** Sensory scores of the chicken meatballs.

Sensory Properties ^1^	CS	QS	Q	CS-Q	QS-Q
Appearance	6.34 ± 1.47 ^a^	6.40 ± 1.56 ^a^	5.23 ± 1.70 ^b^	5.31 ± 1.75 ^b^	5.37 ± 1.83 ^b^
Color	5.97 ± 1.40 ^a^	6.09 ± 1.60 ^a^	5.66 ± 1.85 ^a^	5.69 ± 1.83 ^a^	5.91 ± 1.79 ^a^
Flavor	5.89 ± 1.59 ^a^	5.91 ± 1.70 ^a^	5.34 ± 1.41 ^a^	5.60 ± 1.75 ^a^	6.09 ± 1.52 ^a^
Taste	6.03 ± 1.44 ^a^	5.83 ± 1.56 ^a^	4.69 ± 1.62 ^b^	5.43 ± 1.63 ^ab^	5.97 ± 1.87 ^a^
Texture	5.57 ± 1.72 ^ab^	5.60 ± 1.63 ^ab^	5.23 ± 1.85 ^b^	5.71 ± 1.66 ^ab^	6.17 ± 1.99 ^a^
Overall acceptability	5.77 ± 1.54 ^a^	5.89 ± 1.53 ^a^	4.77 ± 1.44 ^b^	5.80 ± 1.51 ^a^	6.46 ± 1.77 ^a^

^1^ Evaluated on a nine-point hedonic scale from 1 = disliked extremely to 9 = liked extremely. CS, corn starch; QS, quinoa starch; Q, quinoa seed; CS-Q, corn starch and quinoa seed; QS-Q, quinoa starch and quinoa seed. Values are expressed as mean (*n* = 35) ± standard deviation. ^a,b^ Mean values with different letters in the same row are significantly different according to Duncan’s multiple range test at *p* < 0.05.

## Data Availability

Data was generated during the study.
